# *Cutibacterium acnes* as an Opportunistic Pathogen: An Update of Its Virulence-Associated Factors

**DOI:** 10.3390/microorganisms9020303

**Published:** 2021-02-02

**Authors:** Constance Mayslich, Philippe Alain Grange, Nicolas Dupin

**Affiliations:** 1 NSERM Institut Cochin, INSERM U1016-CNRS UMR8104, Equipe de Biologie Cutanée, Université de Paris, 75014 Paris, France; constance.mayslich@inserm.fr (C.M.); philippe.grange@aphp.fr (P.A.G.); 2 Service de Dermatologie-Vénéréologie, Groupe Hospitalier APHP.5, CNR IST Bactériennes—Laboratoire Associé Syphilis, 75014 Paris, France

**Keywords:** *C. acnes*, inflammation, innate immunity, virulence factors, characterization

## Abstract

*Cutibacterium acnes* is a member of the skin microbiota found predominantly in regions rich in sebaceous glands. It is involved in maintaining healthy skin and has long been considered a commensal bacterium. Its involvement in various infections has led to its emergence as an opportunist pathogen. Interactions between *C. acnes* and the human host, including the human skin microbiota, promote the selection of *C. acnes* strains capable of producing several virulence factors that increase inflammatory capability. This pathogenic property may be related to many infectious mechanisms, such as an ability to form biofilms and the expression of putative virulence factors capable of triggering host immune responses or enabling *C. acnes* to adapt to its environment. During the past decade, many studies have identified and characterized several putative virulence factors potentially involved in the pathogenicity of this bacterium. These virulence factors are involved in bacterial attachment to target cells, polysaccharide-based biofilm synthesis, molecular structures mediating inflammation, and the enzymatic degradation of host tissues. *C. acnes*, like other skin-associated bacteria, can colonize various ecological niches other than skin. It produces several proteins or glycoproteins that could be considered to be active virulence factors, enabling the bacterium to adapt to the lipophilic environment of the pilosebaceous unit of the skin, but also to the various organs it colonizes. In this review, we summarize current knowledge concerning characterized *C. acnes* virulence factors and their possible implication in the pathogenicity of *C. acnes*.

## 1. *C. acnes* Characteristics

The genus *Cutibacterium acnes* (*C. acnes*, formerly known as *Propionibacterium acnes* or *P. acnes*, see below) is a commensal lipophilic Gram-positive bacterium. *C. acnes* is described as diphtheroid or coryneform because it is rod-shaped and slightly curved with a width of 0.4 to 0.7 µm and length of 3 to 5 µm. Anaerobic bacteria are characterized by their inability to grow on solid media in the presence of atmospheric oxygen. However, *C. acnes* is considered an aerotolerant anaerobe because it possesses enzymatic systems able to detoxify oxygen, allowing it to be sustained on the surface of the skin [1].

Following its isolation [2], *C. acnes* was first included in the genus *Bacillus* as *Bacillus acnes,* and then in the genus *Corynebacterium* as *Corynebacterium acnes* or “anaerobic *corynebacteria*” because of its morphology. Based on its ability to produce propionic acid via its anaerobic catabolism, it was then assigned to the genus *Propionibacterium,* subsequently renamed *Cutibacterium*. Genus *Cutibacterium* belongs to a branch of *Actinobacter* and can be split into two groups, one containing the so-called “classic or dairy” species, bringing together saprophytic species isolated from non-human-pathogenic dairy products, and the other containing commensal “skin” species, most found on the surface of human skin. Classic species, such as *Propionibacterium freundenreichii*, which is essential for the ripening of Swiss cheeses, or *Propionibacterium acidipropionici*, known for its beneficial effects in the bovine rumen, have been studied in considerable detail due to their importance to the agri-food industry. By contrast, the pathophysiology of cutaneous species is less well understood.

**Figure 1:**
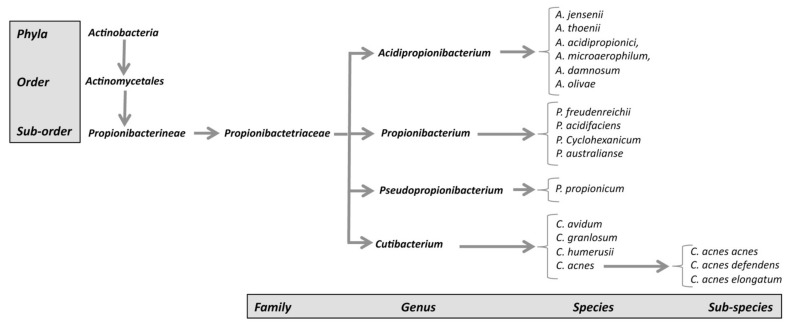
New classification of *C. acnes* strains.

Phylogenetic and genome analyses of classic and cutaneous species have highlighted differences in the 16S RNA gene sequences and the core genome between species. This finding led to proposals to reclassify many species into four different genera:

(1) The genus *Propionibacterium*, comprising the species *P. freundenreichii*, *P. cyclohexanicum*, *P. acidifaciens*, and *P. australianse*.
(2) The new genus *Acidipropionibacterium*, comprising the species *A. jensenii*, *A. thoenii*, *A. acidipropionici*, *A. microaerophilum*, *A. damnosum*, and *A. olivae*.
(3) The new genus *Pseudopropionibacterium,* containing a single species: *P. propionicum*.
(4) The new genus *Cutibacterium*, comprising cutaneous *Propionibacterium* bacteria belonging to the species *C. acnes*, *C. avidum*, *C. granulosum*, and *C. humerusii*. *P. acnes* has thus been renamed *C. acnes* [3]. Moreover, the genus *C. acnes* has been further subdivided into subspecies, such as *C. acnes* subsp. *defendens* [4,5], and *C. acnes* subsp. *elongatum* [6] (Figure 1).

Unlike other Gram-positive bacteria, *C. acnes* has a unique cell wall and envelope, containing phosphatidylinositol, triacylglycerol, and many other common lipids [7]. The cell wall of *C. acnes* consists of peptidoglycan (PNG), but of a type different from that of other Gram-positive bacteria, in that the peptide chain contains the L-acid L-diaminopelic acid and D-alanine. Analyses of *C. acnes* lipoglycans have also revealed the presence of a lipid anchor based on fatty acids and shown that the polysaccharide moiety contains significant amounts of mannose, glucose, and galactose, together with an amino sugar thought to be a diaminohexuronic acid [8]. *C. acnes* has been shown to survive for long periods of time in human tissues with a low oxidative potential. Indeed, *C. acnes* has all of the proteins required for oxidative phosphorylation (NAPDH dehydrogenase/complex I, cytochrome *c* reductase, cytochrome *c* oxidase, and FoF1-type ATP synthase). It also possesses the cytochrome *d* oxidase genes, enabling it to grow in conditions in which limited amounts of oxygen are present and to tolerate oxygen for a few hours. Despite its tolerance of the presence of air, *C. acnes* cannot be reliably detected by culture in aerobic conditions due to its very slow growth (5 to 7 days), associated with a division time of about five hours [9]. A limited number of *C. acnes* strains possess a linear plasmid carrying several genes, including a locus for tight adherence (tad) encoding adhesive Flp (fimbrial low-molecular weight protein) pili [10]. Antibiotics were found to be effective against inflammatory acne, but the selection pressure exerted by antibiotic treatment for this condition has led to the induction of antibiotic resistance in up to 40% of *C. acnes* strains (resistance to erythromycin, clindamycin, and tetracycline), increasing the likelihood of treatment failure [11]. Interestingly, it should be noted that the anaerobic species may have intrinsic resistance due to a lack of molecular mechanisms involved in the uptake of the antibiotic [1]. Therefore, antimicrobial susceptibility testing on *C. acnes* strains should be implemented largely in order to adapt antibiotic treatments and to obtain epidemiological data on *C. acnes* antibiotic resistance. In this review, we summarize current knowledge concerning characterized *C. acnes* virulence factors and their possible implication in the pathogenicity of *C. acnes*, which may interest researchers and clinicians investigating the physiopathology of *C. acnes*.

## *2. C. acnes* Classification

*C. acnes* strains were previously classified into two main types, I and II, on the basis of their cell wall carbohydrate content and serum lectin responses [12,13]. It was then suggested that *C. acnes* strains could be distinguished on the basis of random amplification of polymorphic DNA (RAPD) analysis [14]. Analyses of the sequences of the non-ribosomal housekeeping genes *RecA* and *tly,* and the use of the QUBPa1 and QUBPa2 mAbs, specific for type I and II strains, respectively, subsequently showed that the two types corresponded to evolutionary lineages of *C. acnes* displaying both genetic and phenotypic differences. An additional phylotype, type III, corresponding to strains with filamentous appendages, was then added to the classification [15,16]. Based on 16S rRNA gene analysis, *C. acnes* strains were divided into ribotypes (RTs), which could be used to differentiate between types I, II, and III, associated with healthy skin or acne [17]. RT4, RT5 and RT8 appeared to be found in acne lesions, whereas RT2 and RT6 were mostly found on healthy skin [18]. Multi-locus sequence typing (MLST) and single-locus sequence typing (SLST) methods have been developed for this species, to increase typing resolution. Depending on the number and nature of the housekeeping genes selected, two schemes for discriminating between *C. acnes* strains have been described. The Belfast scheme, using seven target genes, can differentiate type I into clades IA_1_, IA_2_, IB, and IC [19,20]. A multiplex touchdown PCR typing method has also been developed that can discriminate between the IA_1_, IA_2_, IB, IC, type II, and type III phylotypes, providing a rapid overview of the *C. acnes* types present in a population [21]. The other scheme, the Aarhus scheme, uses nine target genes to split type I into clades I-1a, I-1b, and I-2 [22]. This scheme appears to be the most discriminant of these two schemes [23]. SLST and whole-genome sequencing (WGS) methods were subsequently used to develop a new scheme capable of differentiating *C. acnes* strains into clades IA-1, IA-2, IB-1, IB-2, IB-3, IC, II, and III [24], but some *C. acnes* strains were not identified with this scheme [25] (Table 1). MS-based typing for routine analysis has been evaluated with the MALDI-TOF MS method, to characterize *C. acnes* strains in diverse samples [26]. This method is very useful for rapidly discriminating *C. acnes* phylotypes, although missing some of them [26]. However, this approach was improved using profiling of identified biomarkers, such as ribosomal subunit proteins, and named MALDI-MS prototyping, which allowed discrimination of all of the *C. acnes* phylotypes. Although this method needs to ultrafiltrate bacteria whole pellets in order to analyze the concentrated protein fraction, it is nonetheless compatible with the analysis of a large number of *C. acnes* strains [27]. Another attempt to facilitate *C. acnes* typing involved the use of the MLVA method to analyze the polymorphism of 13 VNTRs; the results of this method were well-correlated with those for the MLST and SLST methods [28]. Nomenclatures highlight the need for standardization. WGS is of potential interest for this purpose because it should provide a higher-resolution phylogeny more rapidly and at lower cost [29,30].

## 3. Skin Microbiota and Acne

Human skin consists of a stratified, cellular epidermis and an underlying dermis of connective tissue. The epidermis is characterized by a stratified squamous epithelium composed of about 80–90% keratinocytes, which progressively move from the epidermal basement membrane towards the surface of the skin, forming several well-defined layers during this transit. Other cells resident within the epidermis include melanocytes, Langerhans’ cells, and Merkel cells. The epidermis has several very important functions in the protection of the body against environmental hazards, and it also acts as an immunological barrier, modulating the microbial population of the skin [32,33,34]. The dermis is characterized by a layer of fibroblast cells that also contains immune cells, such as macrophages, neutrophils, and resident T cells.

Acne vulgaris is a chronic inflammatory skin disease mostly found in the teenage population but also in young adults [35]. In a large majority of cases, acne is not a serious disease but if left untreated, can have serious consequences both physically and psychologically. However, there are severe forms of acne that cause permanent scarring, which may have severe consequences for personality development in young people, leading to social and economic problems. Adolescents suffering from acne are more anxious, socially inhibited, and aggressive than adolescents without acne. Furthermore, acne is the only skin disease for which outcome has been implicated as a risk factor for suicide, particularly in men [36,37].

Acne vulgaris is a disorder affecting the pilosebaceous unit (PSU), resulting in both inflammatory and non-inflammatory clinical lesions. Most patients have a mixture of non-inflammatory comedones and inflammatory papules, pustules, and nodules. Acne ranges in severity from mild symptoms to rare cases of severe rare fulminant infection, with a small subset of patients displaying a highly destructive inflammatory response that is often associated with scarring [38]. The hair follicle or pilosebaceous unit (PSU) groups together sebaceous glands, a hair, and a follicle duct made up of epithelial cells. The sebaceous glands produce sebum which flows into the infundibulum to be released to the surface of the skin. Sebum contains a wide variety of lipids, such as squalene, wax esters, triglycerides, and free fatty acids. The production of sebum is linked to the interaction between the PSU and the thyroid and androgenic hormones, and is dependent on age [39]. Acne occurs mostly in teenagers, in whom hormonal secretion is often imbalanced, leading to an increase in sebum secretion by the sebaceous glands in the PSU [40]. The accumulation of sebum in the infundibulum induces keratinocyte proliferation in the follicular wall, promoting PSU obstruction and the formation of comedones. PSU obstruction leads to hypoxia, favoring the development of *C. acnes*. By interacting with the cells of the PSU, this bacterium then triggers the expression of pro-inflammatory molecules, resulting in strong inflammation. Acne vulgaris thus appears to be a multifactorial disorder, involving sebaceous hyperplasia, follicular hyperkeratinization, and colonization by *C. acnes*, acting as an opportunistic pathogen. *C. acnes* has also been shown to influence the formation of comedones by secreting propionic acid, which modulates keratinocyte differentiation [41].

However, the long-standing hypothesis that acne results from *C. acnes* proliferation has evolved, because this bacterium is also involved in the maintenance of healthy skin and can act as an opportunistic pathogen in various inflammatory conditions, including acne. Amplification- and sequencing-based methods have shed considerable light on the huge diversity of the skin microbial community. Despite the possible interference of sampling methods with the microbiome profile identified [42,43], it has been demonstrated that healthy skin harbors microorganisms from multiple kingdoms: bacteria, fungi, and viruses. Most skin bacteria belong to four phyla: Actinobacteria (Corynebacterineae, Propionibacterineae), Proteobacteria, Firmicutes (Staphylococcaceae), and Bacteroidetes. The composition of the skin microbiota varies between individuals, but *C. acnes* seems to occur mostly at sebaceous sites (on the face, back, and pre-thoracic region), reflecting its ability to survive in anaerobic and lipid-rich conditions. By contrast, *Staphylococcus* and *Corynebacterium* species are mostly found at humid sites, with a more diverse bacterial population occupying dry sites [17,44,45]. *C. acnes* is, by far, the most abundant bacterium in the skin microbiota, and its load does not seem to differ between healthy skin and skin affected by acne. It has been suggested that inflammatory acne is triggered by an imbalance in the skin microbiota associated with the selection of specific types of *C. acnes* [25,45,46]. It has been shown that phylotypes IA-1 are strongly associated with inflammatory acne and acne fulminans. These phylotypes produce high levels of porphyrins, which can promote inflammation [17,18,20,22,38,47]. The population of cutaneous microorganisms should be considered a dynamic feature. Bacteria can interact with each other (*S. epidermidis—C. acnes*) and may also be influenced by changes in host characteristics (hormonal disorders, stress, environmental changes), promoting the selection of pathogenic strains of *C. acnes* capable of producing several virulence factors (biofilm, surface proteins) increasing inflammatory capacity [48,49,50]. The skin also reacts to the commensal microbiota, with effects on the immune response and epidermal development, contributing to skin health and disease [51]. Modulation of the population of *C. acnes* strains on the skin, without inducing a negative reaction, could therefore potentially be used in the treatment of microbiome-related diseases [52].

## 4. *C. acnes* and Other Infections

In addition to its role in inflammatory acne, *C. acnes* is emerging as an important opportunistic pathogen. It has been characterized in many other clinical situations related to inflammatory diseases and implant-associated infections [53,54,55,56]. *C. acnes* is now the second most frequent pathogen, after coagulase-negative staphylococci, isolated from infected internal cerebral ventricular bypasses, and rates of infection with this bacterium have increased from 1.5 to 38% [57]. *C. acnes* is also isolated in blood culture where it may represent up to 80% of the isolated anaerobes. The improvement of growing anaerobic bacteria and the use of MALDI-TOF technology has considerably improved the detection rate, however, the presence of *C. acnes* in blood is considered as a contaminant because the presence of *C. acnes* was not associated with clinically-relevant conditions and, therefore, being introduced during sampling [58,59]. *C. acnes* is also a significant cause in postoperative central nervous system infections (PCNSIs) [60], leading to an increase in hospitalization time and antibiotic treatment [61]. Recently, using *C. acnes* typing and clinical data relative to treatment, the differentiation between a true infection and a contamination was reported [62]. However, more investigations will be necessary to determine the prevalence of *C. acnes* phylotypes in such samples and the nature of virulence factors which may be expressed.

*C. acnes* is found on bone and bone-related implants, and is isolated in more than 50% of osteoarticular prosthesis infections, particularly those involving shoulder prostheses [63,64]. It has been isolated following shoulder arthroplasty, and from the macrophages in the shoulder joint [65,66] and in cases of intervertebral disc inflammation [67]. *C. acnes* has also been isolated from bone and joint infections, and cases of intervertebral disc degeneration [26,68,69], in which it decreases the resorption capacity of osteoclasts [70]. It was recently shown, in an in vivo rat model of subclinical orthopedic device-related infection, that *C. acnes* types II and III can induce osteolysis due to their persistence on the surface of the device [71]. *C. acnes* has also been isolated in cases of breast implant infections associated with the formation of a biofilm causing chronic inflammation [72], in cases of prosthesis endocarditis, from cardiovascular devices, and in cases of pericarditis [73].

Moreover, the pro-inflammatory properties of *C. acnes* may also contribute to sarcoidosis, synovitis-acne-pustulosis-hyperostosis-osteitis (SAPHO) syndrome and prostate cancer [74,75,76,77,78].

Analyses of the different types of *C. acnes* types in these infections have revealed that types IB and II are overrepresented in foreign material and soft tissue infections, bacteremia, and healthy skin. Type II predominates in infections on endodontic material, whereas types IB and IC are more associated with the colonization of prostate, urinary tract, and orthopedic equipment. Finally, phylotype III strains, which are rarely found on the face, account for about 20% of isolates from healthy skin. They are particularly predominant on the trunk and in intervertebral disc infections, and have been implicated in progressive macular hypomelanosis (PMH) [18,20,29,79,80,81].

Controversy remains about whether the isolation of *C. acnes* strains in different tissues, with the exception of skin, should be considered as a true infection or a contamination. *C. acnes* infections are generally considered to be associated with surgery or implantation of foreign devices, particularly when *C. acnes* is isolated from prosthetic joints infections (PJI), where it was first considered as a contamination. Previously it was shown that the predominant phylotypes in PJIs were IB and II, whereas phylotype IA was mostly identified in severe acne, arguing in favor of different strains between skin and PJI [20]. However, it was shown recently that phylotypes IA_2_ and IA_1_ were also identified in PJI samples [82]. Diagnosing PJIs involving *C. acnes* is difficult but the improvement of bacteria culture associated with their molecular identification has led to an increase in their rate of detection. To date, to ascertain *C. acnes*-related PJI true infection, the same *C. acnes* phylotype must be grown in at least two different tissue and/or synovial fluid samples. However, if different *C. acnes* phylotypes are found, it may reflect polyclonal infection or a contamination. Therefore, using *C. acnes* typing systems to characterize strains between samples is necessary. However, obtaining good quality samples, and avoiding contaminations linked to the surgical intervention and its transport, are also highly important. Overall, it appears that the bacteriological data should be combined with the clinical information to provide valuable information to evaluate if the presence of *C. acnes* in PJIs is due to a contamination or a true infection [83].

## 5. *C. acnes* and Inflammation

The PSU consists of many different cells and is in contact with dermal structures. Several target cells (keratinocytes, monocytes, sebocytes, and fibroblasts) come into contact with *C. acnes* during the inflammatory process. All *C. acnes* target cells have been shown to react to stimulation by *C. acnes* and to produce many pro-inflammatory molecules in vitro.

Innate immunity involves the recognition of microbes present on the skin through pattern recognition receptors (PRRs), such as Toll-like receptors (TLRs), NOD-like receptors (NLRs), AIM2-like receptors (ALRs), RIG-I-like receptors (RLRs), C-type lectin receptors (CLRs), protease-activated receptors (PARs), and intracellular DNA sensors (cGAS-STING). TLRs are the most studied of these PRRs in the context of acne. TLRs are transmembrane proteins, and this family of proteins has 11 members (TLR1-TLR11) in humans. They have been classified into two groups, according to whether they are transmembrane proteins located at the cell surface (TLR1, TLR2, TLR4, TLR5, TLR6, and TLR11) or intracellular proteins (TLR3, TLR7, TLR8 and TLR9). They contain two domains: an intracellular Toll-interleukin 1 (IL-1) receptor (TIR) domain that transmits signals triggering the production of pro-inflammatory molecules, and an extracellular domain containing a leucine-rich repeat (LRR). The LRR is involved in the recognition of various cell-wall components, including lipoproteins, lipids, proteins, and nucleic acids, in the form of pathogen-associated molecular patterns (PAMPs) or damage-associated molecular patterns (DAMPs). TLR4 mostly acts together with CD14 in the recognition of Gram-negative bacteria via lipopolysaccharide (LPS). By contrast, TLR2, in association with TLR1 or TLR6, has a more extensive spectrum and can recognize lipoproteins, in addition to peptidoglycan (PGN), lipoteichoic acid (LTA), lipoarabinomannan, and zymosan, from Gram-positive bacteria and fungi, whereas TLR5 recognizes flagellin from both Gram-positive and Gram-negative bacteria. TLR2 and/or TLR4 can also recognize endogenous ligands or DAMPs, such as hyaluronic acid, oxidized proteins, lipids, and lipoproteins. By contrast, TLR3, TLR7, TLR8, and TLR9 localize to endosomes, where they detect nucleic acids derived from viruses, bacteria, or damaged cells. The roles of TLR10 and TLR11 remain unclear, and TLR10 seems to be able to form dimers with TLR1-2, TLR4, TLR6, and TLR8 [84,85,86,87]. Once the PRRs on the surface of the target cell have been activated by PAMPs or DAMPs, they initiate the innate immune response by producing chemokines, cytokines, and antimicrobial peptides (AMPs).

During inflammatory acne, oxidized sebum lipids induce a cytotoxic inflammatory reaction, which is further exacerbated by the presence of *C. acnes* in the PSU and the chemotaxis of polynuclear neutrophils to the site of inflammation. Moreover, *C. acnes* can release lytic enzymes (metalloproteases, lipases, proteases, hyaluronidases) into its environment, and these enzymes trigger disruption of the follicular epithelium and activation of the immune system [88]. Interestingly, it has recently been shown that IL-36γ plays a key role in EGFRi/MEKi acneiform toxicity, with a number of transcription factors acting simultaneously on the IL-36γ promoter in keratinocytes, including a *C. acnes* signal inducing upregulation of the *IL-36γ* gene [89] and underlying the crosstalk between *C. acnes* and host cells.

*C. acnes* activates the innate immune recognition system mediated via TLR2 and TLR4 and activation of the NF-κB and MAPK signaling pathways, and the NLRP3 inflammasome pathway. *C. acnes* induces production of the proinflammatory molecules IL-1α/β, IL-6, CXCL8/IL-8, IL-12, granulocyte-macrophage colony-stimulating factor (GM-CSF), TNF-α, β-defensin-2 (hBD-2), matrix metalloproteases (MMPs) by keratinocytes, sebocytes, and monocytes in vitro. These pro-inflammatory molecules are also produced ex vivo in acne lesions [90,91,92,93,94,95,96,97,98,99]. Moreover, *C. acnes* produces several extracellular proteases able to trigger the PAR-2 signaling pathway leading to the production of pro-inflammatory molecules, such as IL-1α, CXCL8/IL-8, TNF-α, hBD-2 and MMPs, contributing to amplify the inflammatory reaction [100].

*C. acnes* can also induce the massive production of reactive oxygen species (ROS) by keratinocytes, mediated by the cytoplasmic NADPH oxidase. Stimulation leads to the rapid release of superoxide anions and their conversion into H_2_O_2_, which is further detoxified into water by the GSH/Gpx system. This process requires the scavenger receptor CD36, which induces CXCL8/IL-8 production independently of the TLR2-signaling pathway and may contribute to the amplification of the inflammatory reaction [101]. *C. acnes*-induced ROS also stimulate the NF-κB and MAPK pathways contributing to iNOS/NO and Cox2/PGE2 production in macrophages [102]. The *C. acnes* factor CAMP1 has also been shown to recognize TLR2 and to induce the production of CXCL8/IL-8 by keratinocytes [103]. Moreover, *C. acnes* also stimulates the type I interferon (IFN-I) pathway via the cGAS-STING pathway in macrophages [104].

**Figure 2:**
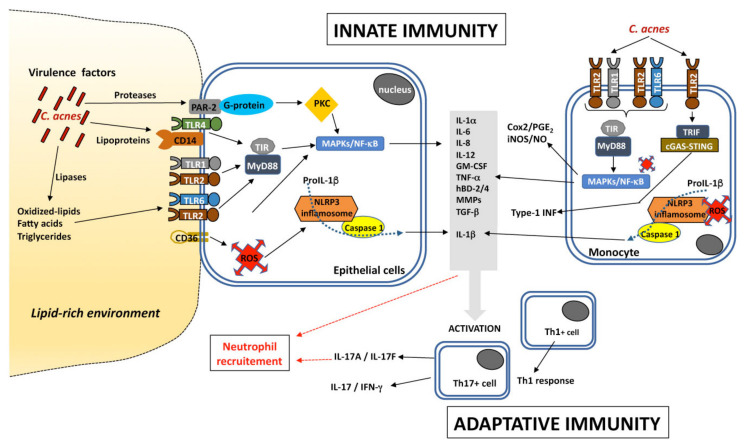
Inflammatory pathways induced by *C. acnes.*

In situ studies of acne lesions have shown that the genes encoding CXCL8/IL-8, hBD-4, MMP1, MMP2, TLR2, and TLR4 are upregulated in the epidermis [93,95], and that this upregulation is associated with the activation of the NF-κB and AP-1 transcription factors [105]. *C. acnes* also plays a crucial role in modulating matrix metalloproteinase (MMP) activity, contributing to both tissue remodeling after inflammation and the scarring process. *C. acnes* increases proMMP2 expression in human dermal fibroblasts via the NF-κB pathway [106]. It has recently been shown that the transcription factor GATA6, which has been implicated in upper PSU homeostasis through control over cell differentiation, is downregulated in the upper PSU in acne, contributing to increase the expression of factors involved in inflammatory acne [107]. *C. acnes* also triggers an adaptive immune response. Indeed, patients with inflammatory acne have been shown to have high serum titers of antibodies against *C. acnes* directed against several putative surface proteins [108]. Moreover, the presence of activated T helper 1 (Th1) lymphocytes has been shown in early inflamed acne lesions [109], and a Th17-related response mediated by the activation of CD4^+^ T cells, leading to the generation of Th17 cells and the secretion of IL-17, whose level appears to be higher with acnes-related strains [110,111] (Figure 2). Overall, *C. acnes* is able to induce inflammatory response by activating several signaling pathways which may involve several virulence factors.

## 6. *C. acnes* Virulence Factors

The *C. acnes* genome was sequenced in its entirety in 2004. It is a single circular chromosome of 2,560,265 base pairs corresponding to 2333 potential genes [112]. During the past decade, several biochemical, transcriptomic, and proteomics analyses have shown that the various phylotypes of *C. acnes* have different inflammatory potentials and express different putative virulence factors.

**Figure 3:**
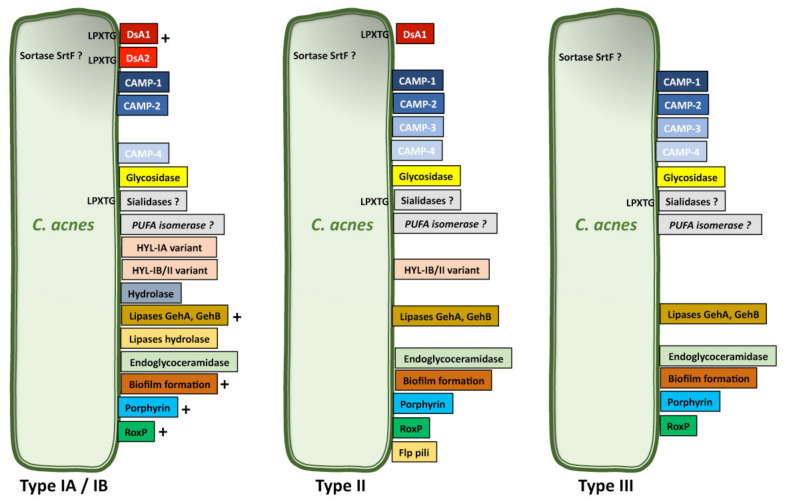
Differential expression of virulence factor by *C. acnes*. Non-exhaustive representation of secreted and/or cell-wall anchored protein. Regarding HylA enzymes, we added both variants and a comment in the figure legend: HYL correspond to the hyaluronate lyase with the HYL-IB/II variant exhibiting a high level of activity and the HYL-IA variant with a lower level of activity. LPXTG correspond to the peptidic sequence recognized by sortase. (+) correspond to highly expressed. (?) the presence in the phylotype is not yet determined. References sources: [108,115,116,117,118].

*C. acnes* has long been considered to be a commensal bacterium, but its implication in various types of infection have led to its emergence as an opportunistic pathogen of low pathogenicity. This pathogenicity, like that of other skin-related bacteria, may be mediated by several molecular mechanisms, including the production of biofilms and the expression of virulence factors triggering immune responses in the host or promoting the adaptation of *C. acnes* to its environment. Various putative virulence factor genes have been identified in the *C. acnes* genome. Some may be involved in cell adhesion, whereas others may mediate inflammation, tissue invasion/degradation in the host, and the synthesis of capsule polysaccharides. These putative virulence factor genes encode sialidases, neuraminidases, endoglycoceramidases, adhesins, thermal shock proteins, CAMP factors, lipases/esterases, and lipases [112,113]. Transcriptomic analyses have revealed that genes encoding putative virulence factors, such as adhesin dermatan-sulfate (DsA1 and DsA2), CAMP factors, polyunsaturated fatty acid isomerase, HtaA iron acquisition protein and GehA lipase, and heat shock proteins, such as HSP20, DnaK, DnaJ, GrpE, and GroEL, are strongly expressed [114,115]. Many studies have, therefore, focused on identification and characterization of the molecular structures secreted by and/or attached to the surface of *C. acnes* and potentially involved in its pathogenicity (Figure 3).

### 6.1. Lipases

Lipase are the enzymes responsible for metabolizing sebum and releasing free fatty acids (FFAs) in the PSU, in addition to several different triglycerides [119,120]. Lipids have been shown to affect the ability of *C. acnes* bacteria to adhere to each other and to surfaces, and one of the functions of the *C. acnes* lipase appears to be enhancing colonization of the PSU by promoting the adhesion of cells to lipid-related components [121]. A triacylglycerol lipase (glycerol-ester hydrolase A: GehA) secreted by *C. acnes* has been identified, together with GehB, which appears to be present in large amounts in human sebaceous follicles [116,122,123]. The genome of *C. acnes* encodes at least 12 putative lipases, including GehA and GehB, both of which carry a signal peptide, consistent with their secretion. However, three of the lipases identified carry a Leu-Pro-X-Thr-Gly (LPXTG) motif at the C-terminus, suggesting that they may be located on the surface of the bacterial membrane [108]. Lipases, including GehA in particular, were the first molecules identified as putative *C. acnes* virulence factors, because they can generate FFAs through the degradation of sebum lipids, thereby promoting inflammation. It has recently been shown that *C. acnes* phylotype IA_1_, which is related to acne-associated strains, is more able to grow on a sebum-like medium, with lipase expression, than a commensal strain not associated with acne [124]. However, GehA is present in all *C. acnes* phylotypes [117], and both GehA and GehB are expressed by *C. acnes* in healthy skin and in the skin of patients with acne [123]. A recent structural analysis of type II *C. acnes* lipase revealed that the active core domain of this protein is shielded by a hydrophobic lid domain, and that its activity is dependent on lipid levels [125]. Lipases appear to play a determinant role in the growth of *C. acnes* in lipophilic environments. Their conformational structure may depend of the lipid level inside the PSU and could influence the pathogenicity of the strain.

### 6.2. Polyunsaturated Fatty Acid Isomerase

The polyunsaturated fatty acid (PUFA) isomerase from *C. acnes* is a yellow 424-residue monomeric protein that can catalyze the isomerization of conjugated linoleic acid (CLA). CLA and its isomers regulate several functions in humans and are present in low levels in food. Six PUFAs have been identified in *C. acnes* and appear to be good candidates for the development of a recombinant isomerase capable of isomerizing CLA [126].

This enzyme has been identified in *C. acnes* strains but its role and pattern of expression in the various phylotypes remain unknown [127].

### 6.3. Hyaluronate Lyase

Hyaluronate lyase (HYL) is an enzyme that degrades hyaluronic acid (HA) and other glycosaminoglycans (GAG), such as chondroitin-4-sulfate, chondroitin-6-sulfate, and dermatan sulfate, present in the extracellular matrix of the epidermis and dermis. The HYL of *C. acnes* has been characterized and sequenced [128,129,130]. Proteomic analyses have shown that HYL is secreted by type IB and type II bacteria [117], and that *C. acnes* produces two HYL variants. The HYL-IB/II variant has a high level of activity and can degrade the HA present in type IB and II strains completely; the HYL-IA variant has a lower level of activity and can only partially degrade the HA present, in type IA strains only [118]. This difference in expression between *C. acnes* strains may account for differences in tissue invasion between phylotypes. Indeed, type AI strains are found mostly on the surface of the skin in inflammatory acne, whereas type IB/II strains are more frequently associated with deep soft tissue infections [18]. It is considered to act as a virulence factor by facilitating the bacterial invasion of tissues and degrading the compounds of the upper layers of the skin and the extracellular matrix, thereby promoting the spread of inflammation. In addition, the products of HA degradation by HYL may be used as nutrients by the bacterium, but may also contribute to inflammation [131,132].

### 6.4. Glycosidase

Glycosidase enzymes break down the carbohydrate and/or glycan structures present on eukaryotic host glycolipids and glycoproteins and can be classified into two groups. The first group contains the exo- and endoglycosidases responsible for hydrolyzing neutral sugars. The second group of glycosidases, sialidases, break down the negatively charged sialic acid (or neuraminic acid). Bacteria have developed enzymatic systems capable of breaking down and using carbohydrates [133]. The *C. acnes* genome contains several genes encoding extracellular or membrane-bound glycosidase. In particular, O-glycan-degrading enzymes have been identified. These enzymes hydrolyze the O-linked glycans present on glycolipids. Two genes corresponding to endoglycoceramidases have also been identified and the enzymes are secreted in all *C. acnes* phylotypes [117,123]. A gene encoding an endo-α-*N*-acetylgalactosaminidase has been cloned (EngPA) and the purified enzyme was shown to hydrolyze core substrates, such as Galβ1,3GalNAcα1*p*NP and GlcNAcβ1,3GalNAcα1*p*NP [134]. A novel exo-β-1,4-mannosidase targeting the β-1,4 glycoside bond between the GlcNac and Mannosyl residues on N-glycans has recently been characterized. This enzyme is encoded by a gene from a carbohydrate processing locus embedded in a gene cluster encoding glycoside hydrolases (GHs) and also containing a gene for an *N*-acetylhexosaminidase involved in the host N-glycan degradation pathway of *C. acnes* [135]. To date, the role of these enzymes in *C. acnes* pathogenicity is still unknown.

### 6.5. Sialidase

Sialidases are enzymes that remove sialic acid from sialoglycoconjugates. The *C. acnes* genome contains three genes encoding sialidases/neuraminidases, two of which have LPXTG motifs for cell wall-anchoring in their C-terminal sequences [112]. The *C. acnes* sialidase appears to be highly immunogenic, and the immunization of mice with this protein elicits the production of an anti-sialidase antibody capable of neutralizing the cytotoxicity of the bacteria [136]. These findings were confirmed for a new *Escherichia coli* vector-based vaccine [137]. However, unlike the glycanases of *C. acnes*, some of which, including endoglycoceramidases in particular [123], have been shown to be secreted, the sialidases of this bacterium are not secreted [117]. Many microorganisms produce sialidases for the purposes of pathogenicity. Recombinant *C. acnes* sialidase can increase *C. acnes* cytotoxicity and adhesion but no study has yet investigated the presence of the anchored sialidases in the cell wall and their role in *C. acnes* pathogenicity.

### 6.6. RoxP

The radical oxygenase of *C. acnes,* RoxP, is an enzyme responsible for reducing free radicals. This ROS-scavenging enzyme was purified from *C. acnes* for the first time in 2016. It is present only in *C. acnes* and is secreted into the supernatant culture. Proteomic analysis revealed the presence of RoxP in phylotypes IA, IB, II, and III [117], but at higher levels in phylotype I than in phylotypes II and III [116] (Figure 3). RoxP binds heme, but further studies are required to determine whether this enzyme makes use of host heme. Although recombinant RoxP has recently been shown to protect skin cells against oxidative stress [138], RoxP is currently thought to help *C. acnes* to survive in oxygen-rich environments, such as the skin surface [139].

### 6.7. Sortase F

Sortases are enzymes capable of covalently attaching various proteins, including adhesion factors, to the cell walls of Gram-positive bacteria. They can be classified into six classes (A–F) on the basis of their amino acid sequences and structures. They recognize the LPXTG peptide sequence and catalyze the transport of the protein, which is produced in the cytoplasm, to the wall. *C. acnes* has only one sortase, a class F enzyme, SrtF, with an activity very similar to that of SrtA from *Staphylococcus aureus*. Interestingly, many of the proteins produced by *C. acnes* possess a sortase recognition sequence, including DsA1 and some sialidases [140], arguing in favor of the presence of protein on the surface of *C. acnes*.

### 6.8. Porphyrin

Porphyrins are fluorescent molecules produced by both eukaryotic and prokaryotic cells. Coproporphyrin III is present in large amounts in acne lesions [141], and type I *C. acnes* strains produce significantly more porphyrins than other phylotypes. Vitamin B12 supplementation increases porphyrin production in acne-associated strains (IA-2, IB-1, and IC), and acne patients tend to have higher serum vitamin B12 concentrations than individuals without acne [47,142]. Vitamin B12 supplementation may affect the metabolome of *C. acnes* in acne patients, disturbing the balance of the skin microbiota [143]. Moreover, the type II *C. acnes* strains associated with healthy skin produce only low levels of porphyrins and carry a *deoR* gene that represses porphyrin biosynthesis [47]. Porphyrins contribute to the perifollicular inflammatory reaction through their cytotoxic effects by stimulating the expression of pro-inflammatory molecules, such as CXCL8/IL-8 and prostaglandin E_2_(PGE_2_), by keratinocytes, and inducing the aggregation of *Staphylococcus aureus* cells and the formation of biofilms in the nose [144]. The acne-related *C. acnes* strains appear to be those sensitive to vitamin B12 and capable of producing large amounts of porphyrin, which appears to be a virulence factor able to induce inflammation.

### 6.9. Biofilm

Bacteria can form biofilms, which provide the bacteria with a resistance to certain antibiotics not observed in the planktonic form. Biofilms are a bacterial extracellular matrix composed of polysaccharides, proteins, and/or extracellular DNA produced endogenous and exogenously [145]. The *C. acnes* genome contains three separate gene clusters encoding enzymes (glycosyltransferases, uridine diphosphate *N*-acetylglucosamine 2-epimerase) involved in polysaccharide synthesis [112]. The *C. acnes* biofilm contains mostly poly-β(1-6)-*N*-acetylglucosamine (PNAG) polysaccharides, proteins, including the GroEL chaperonin, the elongation factors EF-Tu and EF-G, and many enzymes. However, its composition may differ between strains and the results of analyses may depend on the methods of biofilm extraction used [146,147]. Various studies have demonstrated biofilm production by *C. acnes*, particularly on orthopedic material or cardiac devices, and in vitro, using clinical isolates of *C. acnes* from prosthetic hip implants in which biofilm formation was prevented with antibiotics [146,148,149,150]. Studies of a polymicrobial biofilm containing *C. acnes* and *Candida albicans* have shown that *C. albicans* has a protective effect, reducing mortality by 40% of *C. acnes* strains [151]. It is possible that this protection may also extend to other microorganisms within the biofilm, although this has not been demonstrated. Interestingly, in vitro biofilm formation appears to depend on *C. acnes* phylotype rather than the anatomical site of isolation, with type IA_1_ strains producing larger amounts of biofilm than type IA_2_, IC, and II strains [152] (Figure 3). Biofilms are also formed in the pilosebaceous unit and may be responsible for sticking the keratinocytes together, leading to comedone formation and increasing the adhesion of *C. acnes* to the walls of follicle. It should be noted that *C. acnes* biofilms are more frequently observed in patients with acne [153,154] where the biofilm contains several proteins, including CAMP1 implicated in inflammation [155,156] and the miR-146a miRNA, which downregulates the production of pro-inflammatory compounds, such as IL-6, IL-8, and TNF-α in keratinocytes [157]. Moreover, *C. acnes* is capable of invading human bone marrow-derived mesenchymal stem cells and switching from a commensal lifestyle to that of an opportunistic pathogen, by increasing biofilm formation [158].

### 6.10. Pili/fimbriae

Many bacteria express protein appendages on their surface for interaction with the host cell for the purposes of adhesion. *C. avidum* and *C. granulosum* produce exopolysaccharide (EPS)-like structures, whereas *C*. acnes does not, suggesting a divergence of these species during evolution [159]. However, *C. acnes* type II strains isolated from prostate cancers express a putative pilin subunit of adhesive Flp pili, as revealed by the visualization of cell appendages in electron microscopy [10] (Figure 3)**.** Such structures may be involved in the *C. acnes* adhesion process and facilitate its colonization.

### 6.11. DsA1

The proteins present at the microbial surface that can recognize the components of the extracellular matrix (ECM) are called molecular surface components recognizing adhesive molecules of the matrix (MSCRAMM). They have been shown to be involved in the pathogenicity of several skin-associated pathogenic bacteria. Adhesion proteins are of particular interest because of their pathogenic potential and their role in biofilm formation. Nine putative adhesion protein genes have been identified in the *C. acnes* genome. One of these genes encodes the DsA1 protein, which has a LPXTG call-wall anchoring motif, a signal peptide cleavage site (QAEA), and a PTRP sequence repeat [112,113]. However, DsA1 appears to be both secreted and cell-wall anchored, because this protein has no LPXTG motif in some strains [108]. It is produced in the IA-1, IA-2, IB-1, IB-2, and II phylotypes of *C. acnes*, and induces a strong antibody response in acne patients [108,116] (Figure 3). It is principally expressed by *C. acnes* in acne lesions [123]. DsA1 has been shown to bind dermatan sulfate [19] and has also been characterized as a fibrinogen-binding protein. It appears to be highly glycosylated and contains an *N*-acetylgalacosamine (GlaNAc) residue. Like many other fibrinogen-binding proteins from Gram-positive bacteria, DsA1 may be involved in adaptive capacity, promoting the clumping action with fibrinogen and enabling the bacteria to colonize and tolerate the PSU environment [160]. Indeed, studies of plasma proteins have shown that fibrinogen adheres to the surface of *C. acnes* and mediates platelet aggregation [161]. DsA1 therefore appears to be an important surface protein expressed by *C. acnes* and further investigations of its role as a virulence factor are required.

### 6.12. CAMP Factors

**Figure 4:**
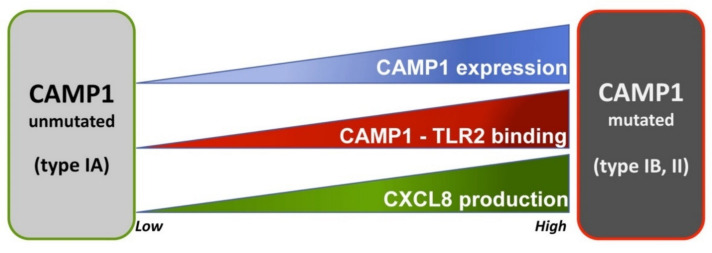
Relationship between CAMP factor 1 polymorphism and inflammation.

Christie–Atkins–Munch–Petersen (CAMP) factors are toxin proteins that form pores in host membranes, leading to host tissue damage. They were first described in a synergic interaction between CAMP factor and the β-toxin (sphingomyelinase C or ASMase) of *Staphylococcus aureus* inducing pore formation in the membrane of sheep erythrocytes and resulting in the lysis of these cells [162]. Five CAMP factor genes have been identified in *C. acnes.* They display partial sequence identity with the gene for the CAMP factor of group B strains of *Streptococcus agalactiae,* and have been named CAMP1 to CAMP5 [112]). The administration of CAMP factor to rabbits and mice has been shown to result in cytotoxicity and lethal effects [163], but synergy between the CAMP factor of *C. acnes* and the ASMase of another bacterium is unlikely to be essential for the virulence of *C. acnes,* because *C. acnes* has been shown to be able to use the host ASMase to increase its virulence [164]. However, it has been shown that CAMP2 gene knockdown in a *C. acnes* strain attenuates the co-hemolytic reaction whereas ∆CAMP4 did not [165]. CAMP genes appear to be highly conserved among bacteria, suggesting that they may play an important role in the pathophysiology of *C. acnes*. Indeed, a phylogenetic analysis of *C. acnes* strains, including both housekeeping and *camp* genes, discriminated effectively between the main *C. acnes* phylotypes, suggesting possible co-evolution with housekeeping genes [16]. CAMP1 and CAMP2 are the predominant CAMP factors produced by *C. acnes* strains, at levels differing between phylotypes IA, II, and III (Figure 3). CAMP1 is strongly expressed by phylotype IB and II strains, whereas CAMP2 is mostly expressed by phylotype IA strains. They were identified as secreted and/or cell-wall anchored protein [116]. Moreover, CAMP1 appears to be one of the most strongly expressed *C. acnes* proteins in the pilosebaceous unit [18,117,123,166]. There is still some debate about the distribution of CAMP proteins, but they appear to be either secreted or attached to the cell surface, because CAMP factors have been isolated from both the external medium and from the surface of *C. acnes* [116,117,159]. CAMP1 has a signal peptide but no LPXTG cell wall-anchoring sequence, and further investigations will be required to understand the molecular mechanisms underlying CAMP production by *C. acnes*. CAMP factors stimulate the innate immune system, and CAMP2 neutralizing antibodies significantly decrease the inflammation induced by *C. acnes* in a mouse ear model [167]. Moreover, CAMP1 interacts with TLR2 to mediate the inflammatory response, and the intensity of the CAMP1–TLR2 interaction appears to be linked to *C. acnes* phylotype. *C. acnes* type IA1 strains display a low level of inflammation induction, with weak or no CAMP1–TLR2 binding. By contrast, highly inflammatory strains displaying strong CAMP1–TLR2 generally belong to types IB and II (Figure 4). However, CAMP1 may not be only the virulence factor inducing inflammation because some IA_1_ strains are more inflammatory. CAMP1 also displays a high degree of genetic polymorphism, with 14 strain-specific amino acid changes inducing strong inflammation and the production of a CAMP1 factor strongly recognized by TLR2 [103]. Further studies are underway to evaluate the role CAMP1 in the induction of inflammation and the impact of its polymorphism.

## 7. Conclusions

*C. acnes* is a bacterium capable of surviving at many different sites within the body. Its greatest evolutionary adaptation is its ability to grow in and colonize the PSU, a highly lipid-rich environment. *C. acnes* is generally a commensal bacterium that is weakly immunogenic and therefore tolerated on the skin of the host. However, it can also act as an opportunistic pathogen, suggesting that it may express different antigenic components at its surface that could be involved in the pathogenicity. Indeed, *C. acnes* strains can produce several virulence factors, leading to a strong inflammatory reaction in the skin and in many other internal organs. However, two main questions remain unresolved. First, can a particular *C. acnes* type become more pathogenic in response to environmental changes in relation to the expression of a specific virulence factor? Second, how does the interaction of *C. acnes* with the skin microbiota influence its pathogenicity?

## Figures and Tables

**Table 1:** *C. acnes* phylotypes.

Phylotypes									
*C. acnes subspecies*		WGS/Ribotyping ^a^	SLST ^b^	MLST8 ^c^	CC	MLST9 ^d^	CC	MALDI-TOF ^e^	MLVA13 ^f^
*C. acnes acnes*	IA-1	RT1, RT5	A1-A34	IA_1_	CC1	I-1a	CC18	IA	IAI
		RT532	B1						
	IA-2	RT1, RT4	C1-C5	IA_1_	CC3	I-1a	CC3	IA	IA2
		RT5							
	IB-1	RT8	D1-D5	IA_1_	CC4	I-1a	CC28	IB	IB
			E1-E9				CC31		
	IB-2	RT3, RT16	F1-F14	IA_2_	CC2	I-1b	CC28	IB	
	IB-3	RT1	H1-H8	IB	CC5	I-2	CC36	IB	
	IC	RT5	G1	IC	CC107	/	/	IB(IC)	/
*C. acnes defendens*		RT2, RT6	K1-K25		CC6		CC53		
	II	RT6		II	CC30	II	CC60	II	II
					CC71				
					CC72				
*C. acnes elongatum*	III	RT9	L1-L10	III	CC77	III	CC43	III	III

WGS: whole genome sequencing; CC: clonal complex. ^a^ Ribotyping [17]; ^b^ SLST [3] ^c^ Belfast scheme [19,20]; ^d^ Aarhus scheme [22]; ^e^ MALDI-TOF analysis [31]; ^f^ MLVA_13_ analysis [28].
